# Pharmacopeial quality of artemether–lumefantrine anti-malarial agents in Uganda

**DOI:** 10.1186/s12936-023-04600-8

**Published:** 2023-05-26

**Authors:** Moses Ocan, Loyce Nakalembe, Caroline Otike, Denis Omali, Allan Buzibye, Sam Nsobya

**Affiliations:** 1 Department of Pharmacology and Therapeutics, College of Health Sciences, Makerere University, P.O. Box 7072, Kampala, Uganda; 2 Department of Pharmacology, Soroti University, P.O. Box 211, Soroti, Uganda; 3 Data Department, Joint Clinical Research Centre, Lubowa, P. O Box 10005, Kampala, Uganda; 4 Pharmacokinetics Laboratory Unit, Infectious Disease Institute, College of Health Sciences, Makerere University, P.O. Box 22418, Kampala, Uganda; 5 Department of Pathology, College of Health Sciences, Makerere University, P.O. Box 7072, Kampala, Uganda

**Keywords:** Artemether–lumefantrine, Substandard quality, Pharmacopoeia, Active pharmaceutical ingredient, Malaria

## Abstract

**Background:**

Substandard anti-malarial agents pose a significant challenge to effective malaria control and elimination efforts especially in sub-Saharan Africa. The quality of anti-malarials in most low-and-middle income countries (LMICs) is affected by several factors including inadequate regulation and limited resources. In this study, the pharmacopeial quality of artemether–lumefantrine (AL) in low and high malaria transmission settings in Uganda was assessed.

**Methods:**

This was a cross-sectional study conducted among randomly selected private drug outlets. The AL anti-malarials available in drug outlets were purchased using overt method. The samples were screened for quality using visual inspection, weight uniformity, content assay and dissolution tests. The assay test was done using liquid chromatography–mass spectrometry (LC–MS). The samples were considered substandard if the active pharmaceutical ingredient (API) content was outside 90–110% range of the label claim. Dissolution test was conducted following United States Pharmacopoeia (USP) method. Data was analysed using descriptive statistics and presented as means with standard deviations, frequencies, and proportions. Correlation between medicine quality and independent variables was determined using Fisher’s exact test of independence at 95% level of significance.

**Results:**

A total of 74 AL anti-malarial samples were purchased from high (49/74; 66.2%) and low (25/74; 33.8%) malaria transmission settings. The most common batch of AL was LONART, 32.4% (24/74), with 33.8% (25/74) being ‘Green leaf’. Overall prevalence of substandard quality artemether–lumefantrine was 18.9% (14/74; 95% CI: 11.4–29.7). Substandard quality AL was significantly associated with setting (*p* = 0.002). A total of 10 samples (13.5%) failed artemether content assay test while, 4 samples (5.4%, 4/74) failed the lumefantrine assay test. One sample from a high malaria transmission setting failed both artemether and lumefantrine assay content test. Of the samples that failed artemether assay test, 90% had low (< 90%) artemether content. All the samples passed visual inspection and dissolution tests.

**Conclusion:**

Artemether–lumefantrine agents, the recommended first-line treatment for uncomplicated malaria with APIs outside the recommended pharmacopeial content assay limit is common especially in high malaria transmission settings. There is need for continuous surveillance and monitoring of the quality of artemisinin-based anti-malarials across the country by the drug regulatory agency.

**Supplementary Information:**

The online version contains supplementary material available at 10.1186/s12936-023-04600-8.

## Background

The World Health Organization (WHO) currently recommends use of vaccine (RTS, S/AS01, R: Central repeat region of *Plasmodium falciparum* circumsporozoite protein, CSP, T: T-cell epitopes of the CSP, and S: Hepatitis B surface antigen, HBsAg) in malaria prevention [1]. This in addition to artemisinin-based anti-malarial agents which have remained highly efficacious for malaria treatment, will strengthen malaria eradication efforts [2]. Globally, artemisinin-based agents are the cornerstone of malaria treatment and have contributed to the gains in the fight against malaria [1]. However, malaria treatment especially in sub-Saharan Africa faces several challenges including widespread distribution and use of substandard anti-malarial agents [3]. Use of substandard artemisinin-based agents may jeopardize the gains in the fight against malaria by contributing to development of drug resistance [4, 5]. Substandard medicines are authorized medical products that fail to meet either quality standards, specifications, while falsified medicines are medical products that deliberately or fraudulently misrepresent their identity, composition, or source [6]. Substandard anti-malarial agents are a common problem especially in malaria-endemic regions. A recent meta-analysis revealed that 19% of anti-malarial agents in low- and middle-income countries (LMICs) were substandard or falsified [7]. A report by the WHO showed that 28.5% of anti-malarial agents sampled from six sub-Saharan African countries were non-compliant with quality specifications [6]. In Uganda, a previous study by Bate et al. [8] found over a third, 35% of anti-malarial agents were of substandard quality.

In sub-Saharan Africa, a previous study showed that 3.8%–8.9% of deaths from malaria were due to use of substandard and falsified anti-malarial agents [9]. In Uganda and Nigeria, substandard and falsified anti-malarial agents contribute to substantial malaria burden especially in children under 5 years [10, 11]. A study by Renschler et al. [12] reported that approximately 122,000 deaths among children under-5 years in Africa were associated with consumption of poor-quality anti-malarial agents. Effective malaria treatment requires use of good quality medicines [13]. Poor quality medicines may result in needless morbidity and mortality and can facilitate emergence of drug resistance [14]. Use of substandard artemisinin-based combinations in the treatment of malaria common in sub-Saharan Africa is likely to increase the risk of local emergence of artemisinin resistance [14, 15].

Substandard anti-malarial agents are a recurrent problem especially in low-and-middle income countries (LMICs) due largely to the inadequate capacity to monitor and control medicine quality [3]. The limited laboratory infrastructure coupled with lack of political will further hinders effective regulation of medicine quality. This study thus sought to assess the pharmacopoeia quality of AL anti-malarials in low and high malaria transmission settings in Uganda.

## Methods

### Study design and setting

This was a cross-sectional study conducted in low (Kabale and Mbarara districts) and high (Apac and Tororo districts) malaria transmission settings between June-December 2021. Artemether–lumefantrine drug samples were purchased over the counter from the drug outlets (pharmacies and drug shops). The study samples were analysed from February–March 2022 at the Infectious Disease Institute Clinical Pharmacology laboratory, Makerere University College of Health Sciences. Dissolution test was done in December 2022-to-January 2023 at the department of Pharmacy Pharmaceutical Science Laboratory at Mbarara University of Science and Technology.

### Sample size, drug outlet selection and sampling

The AL samples were collected from private drug outlets in high and low malaria transmission settings. Private drug outlets in this study are defined as for-profit licensed establishments that dispense medicines. In each district (Tororo, Apac, Mbarara and Kabale), a comprehensive list of the available private drug outlets was compiled using the National drug authority register of drug outlets. Both retail and wholesale private drug outlets were included. Two research assistants, a pharmacist (KJ) and nurse (RK) were trained on the study protocol and collected drug samples for the study. Each of the research assistants separately visited different drug outlets in the study districts. At each drug outlet prior to self-identification, the research assistants inquired whether there were any AL anti-malarial agents. After getting confirmation that there were AL agents in the drug outlets, the research assistants then introduced themselves and explained the study and provided approval letters from the Ethics committee, UNCST and district authorities. A written informed consent was obtained from the pharmacist prior to data collection. All the drug outlets that reported stocking AL anti-malarial agents were purposively enrolled into the study and samples purchased. In each drug outlet, a stock card was also obtained, and AL samples were selected from a batch that had not been sampled from previous drug outlets. In addition, AL samples had to have at least 1 year of shelf-life (time to expiry). In each study district, the research assistants moved from one drug outlet to the next until they could no longer get a batch of AL tablets that was not already sampled. For each batch a minimum of 50 tablets (each adult dose is 24 tablets) were collected in their original packaging and stored in polythene bags marked with unique code. A drug collection checklist was then filled to capture information on the date, location of drug outlet, drug outlet type, batch number, brand name, strength (dose), ‘Green leaf’ and package size. The samples were then transported to the Clinical Pharmacology laboratory at Infectious Disease Institute, Makerere University College of Health Sciences for content assay. Dissolution test was done in the Department of Pharmacy Pharmaceutical Science Laboratory at Mbarara University of Science and Technology.

### Visual inspection of packaging materials and tablets

The packaging, insert and individual tablets on each sample were visually inspected and a detailed description recorded on Microsoft Excel spreadsheet. A modified checklist for Visual Inspection of Medicine (TVIM) provided by the International Council of Nurses in partnership with the United States Pharmacopeia (USP) and Military and Emergency Pharmacists Section of the International Pharmaceutical Federation (FIP) was used for inspection. The description included, stated active pharmaceutical ingredients, date of purchase from drug outlets, name of manufacturer, country of origin, batch number, expiry date, number of tablets per packet, brand name, strength (mg/tablet), dosage statement and storage information. The individual tablets for each sample were also visually inspected for colour, texture, size, uniformity of shape, contamination (embedded spots) and smell, markings (scoring and letters), breaks/ cracks/ splits. However, we did not have the original package from the manufacturers for comparison.

The registration status of each sample brand was checked using online human drug register of the national drug regulator (www.nda.org.ug/register).

### Weight uniformity determination

Twenty randomly selected tablets from each AL batch were weighed and recorded in excel spreadsheet. The standard deviation and percentage relative standard deviation (%RSD) of the weight of each tablet was calculated. The sample passed weight uniformity test if the percentage relative standard deviation per batch was within ± 5% [16, 17].

### Content analysis of artemether and lumefantrine in the samples

#### Sample preparation

Seventy-four (74) batches of fixed dose artemether/lumefantrine tablets were analysed in this study. From a batch, 20 tablets were separately weighed, total weight calculated and gently pounded into a fine smooth powder using ceramic motor and pestle. Sample weights from the fine powder were completely dissolved in 0.5% methanol (MERCK 1060182500) to obtain 2 mg/mL stock solutions of artemether and lumefantrine separately in duplicate. From the stock solutions, final working solutions of Active Pharmaceutical Ingredient (API) concentrations of, 8000 μg/L and 2500 μg/L were prepared and used in the analysis of artemether and lumefantrine content respectively for both duplicate samples. The working solutions were vortexed, and supernatant injected into the LC–MS for analysis.

#### Preparation of calibrators and standard solution

Reference standards of artemether and lumefantrine were separately weighed and dissolved in 0.5% formic acid in methanol to prepare each stock solutions of 2 mg/L. Standard curve concentrations of artemether and lumefantrine calibrators and controls covered a calibration range of 2000–10,000 µg/L and 1000–4000 µg/L, respectively. Artemether and lumefantrine standards were run-in duplicate alongside study samples. The average concentration of the standards was used in final content assessment of the APIs.

#### Sample analysis

The content of Active Pharmaceutical Ingredient (API) in each AL sample was determined using an optimized method in liquid chromatography-mass spectrometry (LC/MS). Spectrometric analysis was carried out using ThermoScientific LCQ Fleet ion trap Liquid chromatography-mass spectrometry (LC/MS^n^) model (Thermo Fisher Scientific Inc. 355 River Oaks Pkwy, San Jose, CA 95134) operated by Xcalibur™ software. During method optimization, the analyte Artemether was spiked in methanol at a concentration of 10 ng/mL and the solution was infused in the Mass spectrophotometer at the flow rate of 10 mL/min. During the infusion, the positive monoisotopic mass of artemether (precursor ion) was identified and selected in a single ion monitoring mode. After which, automatic tuning was applied to maximize the MS parameters such as spray voltage, spray current, sheath-gas flow rate, auxiliary gas flow rate, capillary temperature, capillary voltage, source heater temperature and tube lens. Manual optimization of the capillary temperature was done. Then the molecule was bombarded by adjusting the collision energy until the precursor was approximately 10% of the original molecule. The same steps were repeated for lumefantrine analyte. The most abundant transition or fragment was then identified for analysis. After identifying the transition, we then optimized the transition energy. The transition energy, precursor ion and the tuning file were then saved in the LC–MS machine as method. Mass transitions of 163 and 512 were used to detect artemether and lumefantrine analytes, respectively.

The analysis was conducted using Xcalibur LCQ Fleet ion trap system LC/MS system (Thermo Scientific) and separation achieved using Uptisphere 5 µm column (C18-ODB 125 × 2.1 mm, Interchim technology, USA). The column temperature was maintained at 25 °C. The mobile phase was a gradient of eluent A (10 mM; 50:50 Ammonium acetate in methanol and acetonitrile) and eluent B (10 mM; ammonium acetate). The column was conditioned with 70% of eluent A and 30% eluent B before sample injection. The injection volume was 10 µL and the sample run was set at a flow rate of 500 µL/min for a total run time of 9 min. A gradient of 70% eluent A (2 min), 100% (1 min) eluent B then back to 70% eluent A (6 min) to elute the sample through the column.

Using the calibration curves, concentrations of artemether and lumefantrine in each sample were calculated from linear regression analysis of the peak area ratios versus concentration. The linearity was verified using estimates of correlation coefficient (r^2). Quality control was ensured using two levels of quality control samples (QC) (QC-Low = 3000 µg/L and QC-High = 6000 µg/L for artemether and QC-Low = 1500 µg/L and QC-High = 3000 µg/L for lumefantrine). The samples were each processed and analyzed in duplicates, and the average concentration was used to calculate percentage purity of the API in each sample. After all the sample runs, in addition to all samples with substandard API content, we further randomly selected 10% of all AL samples and re-run following the same conditions.

Artemether and lumefantrine United States Pharmacopeia reference standards were purchased from USP (Twinbrook Parkway, MD 20852–1790, USA). Results were expressed as a percentage of the stated amounts of API on the sample label claim. Quality of ACT was assessed by comparing the amount of API detected with the stated label claim and indicated as a percentage of the stated value. The recommended range between 90 and 110% of the stated API content for both artemether and lumefantrine as per United States Pharmacopoeia was used to classify samples as being of acceptable quality [17].

### Dissolution test for the artemether–lumefantrine samples

#### Preparation of dissolution buffer for artemether

For this, 14.2 g of anhydrous disodium hydrogen phosphate and 100 g of sodium lauryl sulfate were accurately weighed and completely dissolved in about 1 L of distilled water. Distilled water was added with continuous mixing to make 10 L of solution. The pH was then adjusted to 7.1 using dilute hydrochloric acid.

#### Preparation of dissolution buffer for lumefantrine

For this, 10 L of 0.1 M hydrochloric acid solution containing 1% benzalkonium chloride was prepared by adding slowly 98 mL of 10.2 M (32%) hydrochloric acid to distilled water with mixing. The pH was then adjusted to 7.1 using sodium hydroxide.

#### Preparation of artemether standard solution and calibration curve

Weigh about 0.050 g of artemether and dissolve in 100 mL of ethanol. Transfer 2 mL of this solution to a conical flask and make up the volume to 100 mL with hydrochloric acid/ethanol (1 mol/L). The flask was then stoppered and place in a water-bath at 55 °C for 5 h. Then the solution was left to cool to room temperature. The absorbance of the solution was then measured using a spectrophotometer in a 1 cm layer cuvette at a wavelength of about 254 nm. From the stock artemether solution, five serial dilutions of artemether analyte ranging from 0.01–0.03 mg/mL were prepared and absorbance of the solutions measured. A calibration curve was then plotted with a slope of R^2^ = 0.999. The calibration curve was used to calculate the concentrations of artemether in solutions of AL test samples.

#### Preparation of lumefantrine standard solution and calibration curve

Weigh about 0.02 g of lumefantrine and dissolve in 100 mL of ethanol. Transfer 2 mL of this solution to a conical flask and make up the volume to 100 mL with hydrochloric acid/ethanol (1 mol/L). The flask was then stoppered and place in a water-bath at 55 °C for 5 h. Then the solution was left to cool to room temperature. The absorbance of the solution was then measured using a spectrophotometer in a 1 cm layer cuvette at a wavelength of about 335 nm. From the stock lumefantrine solution, five serial dilutions of lumefantrine analyte ranging from 0.04 to 0.14 mg/mL were prepared, and absorbance of the solutions measured. A calibration curve was then plotted with a slope of R^2^ = 0.981. The calibration curve was used to calculate the concentrations of lumefantrine in solutions of AL test samples.

#### Procedure for dissolution assay for artemether/lumefantrine tablets

The dissolution test of artemether–lumefantrine tablets was carried out using USP paddle apparatus (DIS 6000 Copley, UK). For the dissolution of artemether from AL tablets, 1000 mL of artemether dissolution buffer was added to each vessel of the paddle apparatus and equilibrated to 37 ± 0.5 °C for 15 min. In each AL batch, a total of six (6) tablets were randomly picked for dissolution test. One AL tablet was placed in each vessel and the setup immediately operated at a paddle speed of 100 rpm for 60 min. After 60 min, 30 mLs of the sample from any three vessels was withdrawn and allowed to cool to room temperature in a beaker and 30 mLs of the sample was replaced in each of the vessels. Dilution of the withdrawn solution for absorbance reading was done using artemether buffer in accordance with Lambert beer’s law. Absorbance of each of the withdrawn solutions was measured using UV-spectrophotometer (6705, Jenway England) at a wavelength of 254 nm against a blank solution of artemether dissolution buffer and mean absorbance used in calculating drug concentration. Percentage artemether dissolution from the AL tablets was calculated using the calibration curve. The recommended specification for artemether dissolution after 60 min is 70% [17].

For the dissolution of lumefantrine from AL tablets, 1000 mL of lumefantrine dissolution buffer was added to each of the vessels of the paddle apparatus and equilibrated to 37 ± 0.5 °C for 15 min. In each AL batch, a total of six (6) tablets were randomly picked for dissolution test. One AL tablet was placed in each vessel and the setup immediately operated at a paddle speed of 100 rpm for 1 h. After 45 min, 30 mLs of sample from any three of the vessels was withdrawn and allowed to cool to room temperature in a beaker and 30 mLs of the sample was replaced in each of the vessels. Dilution of the withdrawn solution for absorbance reading was done using lumefantrine buffer in accordance with Lambert beer’s law. Absorbance was measured using UV-spectrophotometer (6705, Jenway England) at a wavelength of 335 nm against a blank solution of lumefantrine dissolution buffer and mean absorbance used in calculating drug concentration. Percentage lumefantrine dissolution from the AL tablets was calculated using the calibration curve. The recommended specification for lumefantrine dissolution after 45 min is 60% [17].

### Data management and analysis

Data was entered in Microsoft excel and transferred to STATA *ver* 14.0 for analysis. Data on weight uniformity was analysed using mean, relative standard deviation (RSD) and percentage RSD (%RSD). Sample characteristics were summarized using frequencies and proportions. Prevalence of substandard quality was determined using proportions. Correlation between AL quality and independent variables was determined using Fisher’s exact test of independence at 95% level of significance. The sample was classified as substandard if it failed any one of the quality tests.

## Results

### Description of the artemether–lumefantrine samples collected from low and high malaria transmission settings in Uganda

A total of 74 different batches of artemether–lumefantrine (AL) samples were collected. Most, 66.2% (49/74) of the samples were collected from high malaria transmission settings (Tororo district, 44.6% (33/74) and Apac district, 21.6% (16/74). All batches of AL except one, (PA0839K3) collected from Tororo district were registered for use in the country. Majority, 93.2% (69/74) of the samples were from India. Only two samples, 2.7% (2/74) were locally manufactured from Uganda. LONART, 32.4% (24/74) and ARTEFAN, 20.3% (15/74) were the most common brands of artemether–lumefantrine anti-malarial agents in the country (Table 1). Most of the samples, 89.2% (66/74) had standard strength, 20 mg (artemether) and 120 mg (lumefantrine) of the active pharmaceutical ingredients (APIs). Of the nine samples, 12.2% (9/74) that contained a higher strength of APIs, one had three times the standard dose, 60 mg (artemether) and 360 mg (lumefantrine). A third, 33.8% (25/74) of the AL samples were ‘Green leaf’. The samples described as substandard in this study failed pharmacopoeial content assay test.

**Table 1:** Characteristics of artemether–lumefantrine samples collected from high and low malaria transmission settings in Uganda, June–December 2021 (N = 74)

S/no.	Brand name	No. of samples n (%)	Malaria transmission setting	No. of batches	Label claim (AL/mg)		Manufacturer, country of origin
					20/120	40/240	
1	LONART^a^	25 (33.8%)	Low	8	8	0	BLISS GVS Pharma Ltd, India
			High	17	13	1	
2	ARTEFAN^b^	15 (20.3%)	Low	5	5	0	AJANTA Pharma Ltd, India
			High	10	7	1	
3	LUMARTEM^c^	3 (4.1%)	Low	1	1	0	CIPLA Ltd, India
			High	2	1	0	
4	CO-METHER	5 (6.8%)	Low	2	2	0	AGOG Pharma Ltd, India
			High	3	3	0	
5	KOMEFAN 140	1 (1.4%)	Low	0	0	0	MYLAN Laboratories Ltd, India
			High	1	1	0	
6	COMBIART	5 (6.8%)	Low	0	0	0	STRIDES SHASUN Ltd, India
			High	5	5	0	
7	LONART-DS^d^	1 (1.4%)	Low	0	0	0	BLISS GVS Pharma Ltd, India
			High	1	0	0	
8	LUMERAX	1 (1.4%)	Low	0	0	0	IPCA laboratories Ltd, India
			High	1	1	0	
9	LARIACT	3 (4.1%)	Low	0	0	0	SKANT Healthcare Ltd, India
			High	3	3	0	
10	Cach-ART	2 (2.7%)	Low	0	0	0	CACHET Pharma PVT Ltd, India
			High	2	2	0	
11	LUMAREN	2 (2.7%)	Low	2	2	0	RENE Industries Ltd, Uganda
			High	0	0	0	
12	COARTEM	1 (1.4%)	Low	1	1	0	NOVARTIS PHARMA AG, Switzerland
			High	0	0	0	
13	KOMEFAN	1 (1.4%)	Low	0	0	0	MYLAN Laboratories Ltd, India
			High	1	1	0	
14	Not indicated	4 (5.4%)	Low	4	4	0	IPCA laboratories Ltd, India
			High	1	1	0	
15	LUMITER	6 (8.1%)	Low	3	3	0	MACLEODS Pharma Ltd, India
			High	3	3	0	

### Visual inspection, physical assessment, and weight uniformity test

All samples passed visual inspection of the labels (as per USP guidelines) however, we could not confirm this due to lack of original packing material from the manufacturers. From physical examination of the tablets, there was no powder observed on the tablets and all samples passed physical assessment. The majority, 98.6% (73/74) of samples passed weight uniformity test. Only one sample (1.4%, 1/74) collected from a low malaria transmission setting (Kabale district) failed weight uniformity test with percentage relative standard deviation of 5.3%.

### Prevalence of substandard artemether–lumefantrine anti-malarial agents collected from high and low malaria transmission settings in Uganda

Overall, 18.9% (14/74; 95%CI: 11.4–29.7) of artemether–lumefantrine were of substandard quality. Of the 74 AL samples, 13.5% (10/74) failed artemether assay test. Of these, 9 samples had low (< 90%) while one had higher amount (> 110%) artemether content in the AL tablets (Additional file 1). Four samples, 5.4% (4/74) failed lumefantrine assay test. Of these, 2 samples each had low (< 90%) and high (> 110%) lumefantrine content (Additional file 2). All the samples that failed either artemether or lumefantrine assay tests were from high malaria transmission settings (Tororo and Apac districts). One sample from a batch (CHRT21001E) collected from Apac district failed both artemether and lumefantrine content assay test. Over half, 57.1% (8/14) of the brands of AL anti-malarial agents failed either the artemether or lumefantrine content assay test (Table 2). One sample was not registered for use in the country and was considered to have substandard lumefantrine content regardless of the assay test.

**Table 2:** Assay test results of artemether–lumefantrine samples (N = 74) collected from low and high malaria transmission settings in Uganda, June-December 2021

Brand name	Artemether label claim (mg)	Number of samples tested n (%)	Number of samples with artemether content outside pharmacopeial^a^ range (90–110%) n (%)	Number of samples with lumefantrine content outside pharmacopeial^a^ range (90–110%) n (%)
LONART	20/120	20 (27%)	4 (5.4%)	2 (2.7%)
	40/240	1 (1.4%)	0	0
	80/480	3 (4.1%)	0	0
LONART-DS	80/480	1 (1.4%)	0	0
ARTEFAN	20/120	12 (16.2%)	0	0
	40/240	1 (1.4%)	0	0
	60/360	1(1.4%)	0	0
	80/480^c^	1(1.4%)	1(1.4%)	1 (1.4%)
LUMARTEM	20/120	2 (2.7%)	0	0
	80/480	1 (1.4%)	0	0
CO-METHER	20/120	5 (6.8%)	1 (1.4%)	0
KOMEFAN-140	20/120	1 (1.4%)	1(1.4%)	0
COMBIART	20/120	5 (6.8%)	0	0
LUMERAX	20/120	1 (1.4%)	1 (1.4%)	0
LARIACT	20/120	3 (4.1%)	0	1 (1.4%)
Cach-ART^b^	20/120	2 (2.7%)	2 (2.7%)	1 (1.4%)
LUMAREN	20/120	2 (2.7%)	0	0
COARTEM	20/120	1 (1.4%)	0	0
KOMEFAN	20/120	1 (1.4%)	0	0
LUMITER	20/120	6 (8.1%)	1 (1.4%)	0
Not Indicated	20/120	4 (5.4%)	0	0
Total number		74 (100%)	11 (14.9%)	5(6.8%)

^a^WHO and US Pharmacopeia (90–110%)

^b^One batch (CHRT21001E) of this brand failed both artemether and lumefantrine assay test

^c^The sample was not registered for use in the country by the National drug regulator and was classified as substandard regardless of assay results as per the WHO guidelines

### Artemether–lumefantrine dissolution

Dissolution test was done on all the 74 batches of the AL samples. All the samples had artemether content above 70% after 45 min and lumefantrine above 60% after 60 min as per the pharmacopeia limits stipulated in the USP, 2021 [17] (Table 3).

**Table 3:** Dissolution test results of artemether–lumefantrine samples (N = 74) collected from low and high malaria transmission settings in Uganda, June–December 2021

Brand name	Artemether label claim (mg)	Number of samples tested n (%)	Artemether within pharmacopeial limit, 70% Pass/Fail	Lumefantrine within pharmacopeial limit, 60% Pass/Fail
LONART	20/120	20 (27%)	Pass	Pass
	40/240	1 (1.4%)	Pass	Pass
	80/480	3 (4.1%)	Pass	Pass
LONART-DS	80/480	1 (1.4%)	Pass	Pass
ARTEFAN	20/120	12 (16.2%)	Pass	Pass
	40/240	1 (1.4%)	Pass	Pass
	60/360	1(1.4%)	Pass	Pass
	80/480	1(1.4%)	Pass	Pass
LUMARTEM	20/120	2 (2.7%)	Pass	Pass
	80/480	1 (1.4%)	Pass	Pass
CO-METHER	20/120	5 (6.8%)	Pass	Pass
KOMEFAN-140	20/120	1 (1.4%)	Pass	Pass
COMBIART	20/120	5 (6.8%)	Pass	Pass
LUMERAX	20/120	1 (1.4%)	Pass	Pass
LARIACT	20/120	3 (4.1%)	Pass	Pass
Cach-ART	20/120	2 (2.7%)	Pass	Pass
LUMAREN	20/120	2 (2.7%)	Pass	Pass
COARTEM	20/120	1 (1.4%)	Pass	Pass
KOMEFAN	20/120	1 (1.4%)	Pass	Pass
LUMITER	20/120	6 (8.1%)	Pass	Pass
Not Indicated^a^	20/120	4 (5.4%)	Pass	Pass
Total number		74 (100%)	74 (100%)	74(100%)

^a^One batch of samples with not brand name indicate (HWE111219) from a low malaria transmission setting had 68% artemether dissolution

### Correlation between substandard quality artemisinin-lumefantrine and independent variables

Of the ‘Green leaf’ artemisinin-based combinations, 16% (4/25; 95%CI: 5.9–36.6) failed content assay test. Most, 25% (6/24; 95%CI: 11.3–46.5) of the substandard quality samples were LONART brand. Substandard quality AL was significantly associated with setting (*p* = 0.002). Majority, 28.6% (14/49; 95%CI: 17.5–43.1) of the samples from high malaria transmission setting were of substandard quality (Table 4).

**Table 4:** Relationship between substandard AL quality and independent variables

Characteristic	Description	Number of samples, n (%)	Proportion of substandard quality n (%)	95% CI	Fisher’s exact test
Green leaf AL	No	49 (66.2)	10 (20.4)	11.2–34.4	0.761
	Yes	25 (33.8)	4 (16.0)	5.9–36.6	
Brand name	LONART	24 (32.4)	6 (25.0)	11.3–46.5	0.664
	ARTEFAN	15 (20.3)	1 (6.7)	0.8–37.7	
	CO-METHER	5 (6.8)	1 (20.0)	2.0–75.1	
	LUMITER	6 (8.1)	1 (16.7)	1.8–68.6	
	LARIACT	3 (4.1)	1 (33.3)	2.5–90.9	
	Others	21 (28.4)	4 (19.1)	7.0–42.3	
Setting	High malaria transmission setting	49 (66.2)	14 (28.6)	17.5–43.1	0.002
	Low malaria transmission setting	25 (33.8)	0 (0.0)	–	
AL standard strength (20/120 mg)	No	9 (12.2)	1 (11.1)	1.3–54.1	1.000
	Yes	65 (87.8)	13 (20.0)	11.8–31.8	

## Discussion

The findings of this study demonstrate that one fifth of the AL agents contained APIs which were outside the recommended pharmacopeia range. Most of the AL samples with substandard quality failed assay test of a single active pharmaceutical ingredient (API) in the combination. However, one sample had low content (< 90) of both artemether and lumefantrine. This is like findings of a review by Ozawa et al. [7] done in sub-Saharan Africa and reported 19.7% prevalence of substandard anti-malarial agents. The findings of this study are like those of a previous study by Schiavetti et al. [18] done in DRC which reported out of range content of the active pharmaceutical ingredients (artemether and lumefantrine) as the most common form of substandard quality among the anti-malarial agents. The findings of this study were however not similar to that of a study by Belew et al. [19] done in Ethiopia which reported no existence of substandard anti-malarial agents. The current study focused on AL anti-malarial agents in the private sector as opposed to a study by Belew et al. [19], which was done among public facilities only. In the private drug outlets unlike public facilities, the distribution of medicines is by multiple suppliers which coupled with challenges of medicine regulation common in most low- and middle-income countries could explain the variation in the findings. Substandard anti-malarial agents remain a key problem in the fight against malaria [20]. However, little attention is given to the burden and effects of the use of substandard anti-malarial agents in malaria treatment [21]. In most low-and-middle income countries inadequate drug regulation, lack of political will and limited resources are common risk factors for substandard quality anti-malarial agents [22, 23].

Four of fourteen substandard AL anti-malarial agents found in our study were among the quality assured artemisinin-based combination therapies (QAACT), ‘Green leaf’ ACTs distributed under the co-payment mechanism. Co-payment mechanism in the private sector was developed following large-scale piloting of Affordable Medicines Facility-Malaria (AMFm) from 2010 to 2011 [24]. This was intended to ensure continued provision of subsidies and thus potentially increase access and use of quality assured artemisinin-based combination therapies in malaria treatment [24]. However, the findings of this study indicate prevalence of substandard artemether–lumefantrine among the quality assured artemisinin-based combinations in Uganda. This is an indicator of the challenges in assuring quality under the co-payment mechanism. Investing in improving capacity of the national drug regulator to monitor the manufacture and distribution of QAACT in the country is key in ensuring quality of ‘Green leaf’ ACT in the market.

The findings of this study show that all substandard AL anti-malarial agents were from high malaria transmission settings in the country. This is like the findings of a previous study by Hajjou et al. [22] done in sub-Saharan Africa. High demand coupled with ease of access over the counter of anti-malarial agents in the private sector in these settings potentially drive distribution of substandard agents [25, 26]. Additionally, porous borders common in most low- and middle-income countries may contribute to entry and distribution of medicines of unknown quality [26]. This highlights the need to strengthen anti-malarial quality surveillance in the private sector especially in malaria endemic countries.

Artemisinin resistance was recently reported in Uganda in a study by Balikagala et al. [27]. Other studies also confirmed presence of K13 molecular markers of artemisinin resistance among *P. falciparum* parasites across the country [28] and in Rwanda [29]. Since its emergence in different regions within Southeast Asia, delayed artemisinin parasite clearance has not spread to other malaria affected areas [1]. This is an indicator of the role local factors unique to specific geographical regions play in driving the development of artemisinin resistance among malaria parasites [30]. For the current reported artemisinin resistance in Uganda, understanding the local drivers for its development is key in establishing interventions to mitigate widespread emergence across the country. The findings of the current study demonstrate that seven in every ten substandard AL failed the artemether assay test with over 80% of the failed samples having a low API content (< 90%). This is like the findings of a previous study done in Ghana and Togo by Osei-Safo et al. [31]. The low artemether content in the AL agents found in this study could be contributing to the current emergence of artemisinin resistance among *P. falciparum* parasites [32]. This may be worsened by the potential monotherapy due to the low content of some APIs in the artemisinin-based combination.

In this study, half of the AL samples that failed lumefantrine assay test had a low lumefantrine content (< 90%). In the ACT combination, lumefantrine has a longer half-life than artemisinin [33, 34] and thus helps in clearing parasites that survive artemisinin exposure [35]. The use of ACTs with low lumefantrine content in malaria treatment is likely to result in low blood drug level exposing the malaria parasites to sub-therapeutic lumefantrine concentrations. The concentration of the anti-malarial agent to which the parasites get exposed to is a key determinant of cure [15]. Although there has not been any reported malaria parasite resistance to lumefantrine. If resistant parasites encounter sub-lethal concentrations of a slowly eliminated anti-malarial, they will have a survival advantage and multiply faster than sensitive parasites [36]. This is especially important for poor quality artemisinin-based combinations as they risk the spread of resistance to both the affected API and the unprotected partner API [37]. The long half-life of lumefantrine coupled with substandard quality found in this study may contribute to driving emergence of resistance among malaria parasites in the country [36].

The study had some limitations, the use of overt sampling where pharmacy staff were informed of the purpose of the study and a written informed consent obtained prior to purchasing the drug samples. This is likely to present a risk of bias as some of the outlets refused to be sampled. However, this was minimal as only one drug outlet refused to be sampled in Tororo district. Additionally, if drug outlets knew the drug samples which are of poor quality, this would be hidden from the study team. This is unlikely to have affected the study as the medicine stock cards in all the study drug outlets were first reviewed thus providing the study team with insight into all the anti-malarial agents that were present in stock at the time of the study. Additionally, the research assistants first inquired of the present of AL anti-malarial agents in each drug outlet prior to introducing themselves and the study. Optimization of LC–MS can present some challenge where several species are formed in the ionization source and multiple charging can occur. The LC–MS method used in this study was optimized and the conditions for optimum sensitivity, specificity and reproducibility identified with the MS set to detect specific analytes.

## Conclusion

Artemether–lumefantrine anti-malarials that do not meet the quality specifications are prevalent especially in high malaria transmission settings in Uganda. With the recent discovery of artemisinin resistance in the country, there is need for regular surveillance and monitoring of the quality of artemisinin based anti-malarial agents by the drug regulatory agency.

## Supplementary Information

**Additional file 1.** Results of artemether assay content.

**Additional file 2.** Results of lumefantrine assay content.

## Data Availability

The datasets used and/or analyzed during the current study are available from the corresponding author on reasonable request.

## Abbreviations

ACT: Artemisinin-based combination therapy
API: Active pharmaceutical ingredient
AL: Artemether–lumefantrine
K13: Kelch13 propeller gene
QAACT: Quality assured artemisinin combination therapies
AMFm: Affordable medicines facility-malaria
IP: International pharmacopeia
USP: Unites States pharmacopeia
UNCST: Uganda National Council of Science and Technology
WHO: World Health Organization
LMICs: Low-and middle-income countries
