# Supplementary material for: Pharmacopeial quality of artemether–lumefantrine anti-malarial agents in Uganda
Source: Malar J. 2023 May 26;22:165. doi: 10.1186/s12936-023-04600-8 (PMC10214708; doi:10.1186/s12936-023-04600-8)
Supplement: Supplementary file 1 — Additional file 1. Results of artemether assay content. [file 12936_2023_4600_MOESM1_ESM.doc]

**Additional file 1: Artemether assay results of Artemether-Lumefantrine samples (N=74) collected from high and low malaria transmission settings, June -Dec 2021**

| **S/N** | **Id** | **Brand name** | **Batch** | **Label claim (mg)** | **Calculated Artemether AUC (µg/L)** | **Expected**  **Artemether AUC**  **(µg/L)** | **Calculated**  **Artemether**  **content (mg)** | **Percentage purity of Artemether (%)** | **Verdict**  **(90-110%)** |
| --- | --- | --- | --- | --- | --- | --- | --- | --- | --- |
| 1. | T001 | LONART | JIAFM031 | 80 | 7627.8 | 8000 | 76.3 | 95.3 | PASS |
| 2. | T002 | ARTEFAN | PA040030 | 60 | 8315 | 8000 | 62.4 | 103.9 | PASS |
| 3. | T003 | ARTEFAN | PA04020 | 40 | 8103.2 | 8000 | 40.5 | 101.3 | PASS |
| 4. | T004 | ARTEFAN | DJ04301 | 20 | 8498.7 | 8000 | 21.3 | 106.2 | PASS |
| 5. | T005 | LUMARTEM | BA93397 | 80 | 7551.7 | 8000 | 75.5 | 94.4 | PASS |
| 6. | T006 | CO-METHER | T11077 | 20 | 8523.3 | 8000 | 21.3 | 106.2 | PASS |
| 7. | T007 | ARTEFAN | PA15770 | 20 | 75452.4 | 8000 | 18.6 | 93.2 | PASS |
| 8. | T008 | KOMEFAN 140 | 8115428 | 20 | 5214.9 | 8000 | 13.0 | 65.2 | FAIL |
| 9. | T009 | COMBIART | 7244011 | 20 | 7736.7 | 8000 | 19.3 | 96.7 | PASS |
| 10. | T010 | LONART | KIAFM118 | 80 | 8362.4 | 8000 | 83.6 | 104.5 | PASS |
| 11. | T011 | LONART | KIAFM038 | 80 | 7397.2 | 8000 | 73.9 | 92.5 | PASS |
| 12. | T012 | LONART-DS | KIAFM039 | 80 | 7978.4 | 8000 | 79.8 | 99.7 | PASS |
| 13. | T013 | LONART | KIAFL003 | 40 | 7511.3 | 8000 | 37.6 | 93.9 | PASS |
| 14. | T014 | LUMERAX | FWR340131 | 20 | 9111.6 | 8000 | 22.8 | 113.9 | FAIL |
| 15. | T015 | ARTEFAN | DJ042101 | 20 | 7449.9 | 8000 | 18.6 | 93.1 | PASS |
| 16. | T016 | ARTEFAN | DJ014OJ | 20 | 7439.6 | 8000 | 18.6 | 93 | PASS |
| 17. | T017 | LONART | JIAFJ067 | 20 | 8110.8 | 8000 | 20.3 | 101.4 | PASS |
| 18. | T018 | LONART | LRC601 | 20 | 7032.3 | 8000 | 17.6 | 87.9 | FAIL |
| 19. | T019 | LONART | LRC606 | 20 | 7571.9 | 8000 | 18.9 | 94.6 | PASS |
| 20. | T020 | LUMITER | NAA20290A | 20 | 6054.3 | 8000 | 15.1 | 75.7 | FAIL |
| 21. | T021 | LONART | JIAFJ060 | 20 | 6798.3 | 8000 | 17 | 85 | FAIL |
| 22. | T022 | LONART | KIAFJ104 | 20 | 8229.4 | 8000 | 20.6 | 102.9 | PASS |
| 23. | T023 | LONART | KIAFJ020 | 20 | 8184.6 | 8000 | 20.5 | 102.3 | PASS |
| 34. | T024 | COMBIART | 7243724 | 20 | 8064.9 | 8000 | 20.2 | 100.8 | PASS |
| 25. | T025 | COMBIART | 7243852 | 20 | 7493.9 | 8000 | 18.7 | 93.7 | PASS |
| 26. | T026 | KOMEFAN | 3127188 | 20 | 8238.3 | 8000 | 20.6 | 103 | PASS |
| 27. | T027 | ARTEFAN | DJ0790J | 20 | 8223.1 | 8000 | 20.6 | 102.8 | PASS |
| 28. | T028 |  | HWE510488 | 20 | 7261.3 | 8000 | 18.2 | 90.8 | PASS |
| 29. | T029 | ARTEFAN | DJ04101 | 20 | 7281.1 | 8000 | 18.2 | 91 | PASS |
| 30. | T030 | LONART | KIAFJ084 | 20 | 7775 | 8000 | 19.4 | 97.2 | PASS |
| 31. | T031 | ARTEFAN | PA0839K3 | 80 | 7268.9 | 8000 | 72.7 | 90.9 | PASS |
| 32. | T032 | LUMITER | NAA9268B | 20 | 8076.7 | 8000 | 20.2 | 101 | PASS |
| 33. | T033 | LONART | LRC614 | 20 | 7102.7 | 8000 | 17.8 | 88.8 | FAIL |
| 34. | A034 | COMBIART | 72243721 | 20 | 7594.4 | 8000 | 18.9 | 94.9 | PASS |
| 35. | A035 | LARIACT | AR20049 | 20 | 7321.6 | 8000 | 18.3 | 91.5 | PASS |
| 36. | A036 | LARIACT | AR20048 | 20 | 7658.5 | 8000 | 19.2 | 95.7 | PASS |
| 37. | A037 | LARIACT | AR20052 | 20 | 7385.9 | 8000 | 18.5 | 92.3 | PASS |
| 38. | A039 | CO-METHER | T12045 | 20 | 7434.9 | 8000 | 18.6 | 92.9 | PASS |
| 39. | A040 | ARTEFAN | DJ010DJ | 20 | 7609.3 | 8000 | 19 | 95.1 | PASS |
| 40. | A045 | LUMITER | NAA20286A | 20 | 7281.2 | 8000 | 18.2 | 91 | PASS |
| 41. | A042 | LONART | LRC510 | 20 | 7875.9 | 8000 | 19.7 | 98.4 | PASS |
| 42. | A041 | LONART | KIAFJ085 | 20 | 6730.9 | 8000 | 16.8 | 84.1 | FAIL |
| 43. | A043 | LONART | LRC530 | 20 | 7244.7 | 8000 | 18.1 | 90.6 | PASS |
| 44. | A038 | LUMARTEM | ID92982 | 20 | 7477.7 | 8000 | 18.7 | 93.5 | PASS |
| 45. | A044 | COMBIART | 7243727 | 20 | 7509.8 | 8000 | 18.8 | 93.9 | PASS |
| 46. | A043B | LONART | LRC490 | 20 | 7537.4 | 8000 | 18.8 | 94.2 | PASS |
| 47. | A072 | Cach-ART | CHRT21002E | 20 | 4723.7 | 8000 | 11.8 | 59 | FAIL |
| 48. | A073 | Cach-ART | CHRT21001E | 20 | 5422.4 | 8000 | 13.6 | 67.8 | FAIL |
| 49. | A074 | CO-METHER | T13006 | 20 | 5180.6 | 8000 | 13 | 64.8 | FAIL |
| 50. | K004 |  | HWE510481 | 20 | 8679.9 | 8000 | 21.7 | 108.5 | PASS |
| 51. | KOO7 | LUMAREN | 07221 | 20 | 7918.6 | 8000 | 19.8 | 99 | PASS |
| 52. | K008 | LUMITER | NAJ2005B | 20 | 7676.2 | 8000 | 19.2 | 96 | PASS |
| 53. | KOO9 | CO-METHER | T13035 | 20 | 7950.2 | 8000 | 19.9 | 99.4 | PASS |
| 54. | KOO1 | LONART | A1AFJ031 | 20 | 7539.2 | 8000 | 18.8 | 94.2 | PASS |
| 55. | KOO2 | LONART | A1AFJ026 | 20 | 7864.6 | 8000 | 19.7 | 98.3 | PASS |
| 56. | KOO3 |  | HWE111219 | 20 | 7989.4 | 8000 | 20 | 99.9 | PASS |
| 57. | KOO5 | LONART | K1AFJ085 | 20 | 8144 | 8000 | 20.4 | 101.8 | PASS |
| 58. | K006 | LONART | LRC404 | 20 | 7485.4 | 8000 | 18.7 | 93.6 | PASS |
| 59. | K010 | ARTEFAN | PA11971 | 20 | 7731.6 | 8000 | 19.3 | 96.6 | PASS |
| 60. | M020 | LUMAREN | 03121 | 20 | 7689 | 8000 | 19.2 | 96.1 | PASS |
| 61. | M019 | ARTEFAN | PA02931 | 20 | 8213.9 | 8000 | 20.5 | 102.1 | PASS |
| 62. | M018 | ARTEFAN | PA02811 | 20 | 7898.9 | 8000 | 19.7 | 98.7 | PASS |
| 63. | M013 | ARTEFAN | DJ0839G | 20 | 8325.1 | 8000 | 20.8 | 104.1 | PASS |
| 64. | M012 | ARTEFAN | PA034202 | 20 | 8204.3 | 8000 | 20.5 | 102.6 | PASS |
| 65. | M011 | LONART | LRC588 | 20 | 79994.1 | 8000 | 20 | 99.9 | PASS |
| 66. | M021 | LUMARTEM | QK91643 | 20 | 7287.7 | 8000 | 18.2 | 91.1 | PASS |
| 67. | M022 |  | HWE111217 | 20 | 7567.6 | 8000 | 18.9 | 94.6 | PASS |
| 68. | M023 | LONART | A1AFJ026 | 20 | 8124.5 | 8000 | 20.3 | 101.6 | PASS |
| 69. | M024 | LONART | K1AFJ018 | 20 | 8141.1N | 8000 | 20.4 | 101.8 | PASS |
| 70. | M025 | LONART | K1AFJ020 | 20 | 8075.3 | 8000 | 20.2 | 100.9 | PASS |
| 71. | M017 | CO-METHER | T13007 | 20 | 8266.3 | 8000 | 20.7 | 103.3 | PASS |
| 72. | M016 | LUMITER | NAA20167A | 20 | 8103.4 | 8000 | 20.3 | 103.3 | PASS |
| 73. | M015 | LUMITER | NAA20298A | 20 | 8061.6 | 8000 | 20.2 | 100.8 | PASS |
| 74. | M014 | COARTEM | KX118 | 20 | 8082.9 | 8000 | 20.2 | 101 | PASS |
