# Supplementary material for: Pharmacopeial quality of artemether–lumefantrine anti-malarial agents in Uganda
Source: Malar J. 2023 May 26;22:165. doi: 10.1186/s12936-023-04600-8 (PMC10214708; doi:10.1186/s12936-023-04600-8)
Supplement: Supplementary file 2 — Additional file 2. Results of lumefantrine assay content. [file 12936_2023_4600_MOESM2_ESM.doc]

**Additional file 2: Lumefantrine assay results of Artemether-Lumefantrine samples (N = 74) collected from high and low malaria transmission settings, June-Dec 2021**

| **S/N** | **Id** | **Brand name** | **Batch** | **label claim (mg)** | **Calculated Lumefantrine**  **AUC (µg/L)** | **Expected**  **Lumefantrine AUC**  **(µg/L)** | **Calculated**  **Lumefantrine content (mg)** | **Percentage Lumefantrine**  **purity (%)** | **Verdict**  **(90-110%)** |
| --- | --- | --- | --- | --- | --- | --- | --- | --- | --- |
| 1. | T001 | LONART | JIAFM031 | 480 | 2409.8 | 2500 | 462.7 | 96.4 | PASS |
| 2. | T002 | ARTEFAN | PA040030 | 360 | 2487.6 | 2500 | 358.2 | 99.5 | PASS |
| 3. | T003 | ARTEFAN | PA04020 | 240 | 2472 | 2500 | 237.3 | 98.9 | PASS |
| 4. | T004 | ARTEFAN | DJ04301 | 120 | 2728.9 | 2500 | 130.9 | 109.2 | PASS |
| 5. | T005 | LUMARTEM | BA93397 | 560 | 2648.2 | 2500 | 508.5 | 105.9 | PASS |
| 6. | T006 | CO-METHER | T11077 | 120 | 2331.2 | 2500 | 111.9 | 93.3 | PASS |
| 7. | T007 | ARTEFAN | PA15770 | 120 | 2485.4 | 2500 | 119.3 | 99.4 | PASS |
| 8. | T008 | KOMEFAN 140 | 8115428 | 120 | 2359 | 2500 | 113.2 | 94.4 | PASS |
| 9. | T009 | COMBIART | 7244011 | 120 | 2476.6 | 2500 | 118.9 | 99.1 | PASS |
| 10. | T010 | LONART | KIAFM118 | 480 | 2481.7 | 2500 | 476.5 | 99.3 | PASS |
| 11. | T011 | LONART | KIAFM038 | 480 | 2716.8 | 2500 | 521.6 | 108.7 | PASS |
| 12. | T012 | LONART-DS | KIAFM039 | 480 | 2449.9 | 2500 | 470.4 | 98 | PASS |
| 13. | T013 | LONART | KIAFL003 | 240 | 2322.4 | 2500 | 222.9 | 92.9 | PASS |
| 14. | T014 | LUMERAX | FWR340131 | 120 | 2549.6 | 2500 | 122.4 | 101.9 | PASS |
| 15. | T015 | ARTEFAN | DJ042101 | 120 | 2470.4 | 2500 | 118.6 | 98.8 | PASS |
| 16. | T016 | ARTEFAN | DJ014OJ | 120 | 2358.3 | 2500 | 113.1 | 94.3 | PASS |
| 17. | T017 | LONART | JIAFJ067 | 120 | 2282.2 | 2500 | 109.5 | 91.3 | PASS |
| 18. | T018 | LONART | LRC601 | 120 | 2419 | 2500 | 116.1 | 96.8 | PASS |
| 19. | T019 | LONART | LRC606 | 120 | 2465.1 | 2500 | 118.3 | 98.6 | PASS |
| 20. | T020 | LUMITER | NAA20290A | 120 | 2513.8 | 2500 | 100.6 | 120.7 | PASS |
| 21. | T021 | LONART | JIAFJ060 | 120 | 2584.6 | 2500 | 103.4 | 124.1 | PASS |
| 22. | T022 | LONART | KIAFJ104 | 120 | 2383.8 | 2500 | 114.4 | 95.4 | PASS |
| 23. | T023 | LONART | KIAFJ020 | 120 | 2499.8 | 2500 | 120 | 100 | PASS |
| 24. | T024 | COMBIART | 7243724 | 120 | 2620.6 | 2500 | 125.8 | 104.8 | PASS |
| 25. | T025 | COMBIART | 7243852 | 120 | 2356.1 | 2500 | 113.1 | 94.2 | PASS |
| 26. | T026 | KOMEFAN | 3127188 | 120 | 2378.1 | 2500 | 114.2 | 95.1 | PASS |
| 27. | T027 | ARTEFAN | DJ0790J | 120 | 2572.3 | 2500 | 123.5 | 102.9 | PASS |
| 28. | T028 |  | HWE510488 | 120 | 2592.5 | 2500 | 124.4 | 103.7 | PASS |
| 29. | T029 | ARTEFAN | DJ04101 | 120 | 2322.5 | 2500 | 111.5 | 92.9 | PASS |
| 30. | T030 | LONART | KIAFJ084 | 120 | 3007.5 | 2500 | 144.4 | 120.3 | FAIL |
| 31. | T031 | ARTEFAN | PA0839K3 | 480 | 2624.4 | 2500 | 503.9 | 105 | PASS |
| 32. | T032 | LUMITER | NAA9268B | 120 | 2446.6 | 2500 | 117.4 | 97.9 | PASS |
| 33. | T033 | LONART | LRC614 | 120 | 2733.3 | 2500 | 131.2 | 109.3 | PASS |
| 34. | A034 | COMBIART | 72243721 | 120 | 2678.2 | 2500 | 128.6 | 107.1 | PASS |
| 35. | A035 | LARIACT | AR20049 | 120 | 2468 | 2500 | 118.5 | 98.7 | PASS |
| 36. | A036 | LARIACT | AR20048 | 120 | 1849 | 2500 | 73.9 | 88.8 | FAIL |
| 37. | A037 | LARIACT | AR20052 | 120 | 2438.8 | 2500 | 117.1 | 97.6 | PASS |
| 38. | A039 | CO-METHER | T12045 | 120 | 2406.9 | 2500s | 115.5 | 96.3 | PASS |
| 39. | A040 | ARTEFAN | DJ010DJ | 120 | 2392.6 | 2500 | 114.9 | 95.7 | PASS |
| 40. | A045 | LUMITER | NAA20286A | 120 | 2520.6 | 2500 | 120.9 | 100.8 | PASS |
| 41. | A042 | LONART | LRC510 | 120 | 2768.1 | 2500 | 132.9 | 110.7 | FAIL |
| 42. | A041 | LONART | KIAFJ085 | 120 | 2340.6 | 2500 | 112.4 | 93.6 | PASS |
| 43. | A043 | LONART | LRC530 | 120 | 2532.9 | 2500 | 121.6 | 101.3 | PASS |
| 44. | A038 | LUMARTEM | ID92982 | 120 | 2606.9 | 2500 | 125.1 | 104.3 | PASS |
| 45. | A044 | COMBIART | 7243727 | 120 | 2512.5 | 2500 | 120.6 | 100.5 | PASS |
| 46. | A043B | LONART | LRC490 | 120 | 2511.8 | 2500 | 120.6 | 100.5 | PASS |
| 47. | A072 | Cach-ART | CHRT21002E | 120 | 2708.3 | 2500 | 130 | 108.3 | PASS |
| 48. | A073 | Cach-ART | CHRT21001E | 120 | 1877.9 | 2500 | 99.4 | 82.8 | FAIL |
| 49. | A074 | CO-METHER | T13006 | 120 | 2271.5 | 2500 | 109 | 90.9 | PASS |
| 50. | K004 |  | HWE510481 | 120 | 2450.5 | 2500 | 117.6 | 98 | PASS |
| 51. | KOO7 | LUMAREN | 07221 | 120 | 2497.6 | 2500 | 119.9 | 99.9 | PASS |
| 52. | K008 | LUMITER | NAJ2005B | 120 | 2466.2 | 2500 | 118.4 | 98.6 | PASS |
| 53. | KOO9 | CO-METHER | T13035 | 120 | 2532.9 | 2500 | 121.6 | 101.3 | PASS |
| 54. | KOO1 | LONART | A1AFJ031 | 120 | 2383.7 | 2500 | 114.4 | 95.3 | PASS |
| 55. | KOO2 | LONART | A1AFJ026 | 120 | 2578.4 | 2500 | 123.8 | 103.1 | PASS |
| 56. | KOO3 |  | HWE111219 | 120 | 2497.2 | 2500 | 119.9 | 99.9 | PASS |
| 57. | KOO5 | LONART | K1AFJ085 | 120 | 2535.3 | 2500 | 121.7 | 101.4 | PASS |
| 58. | K006 | LONART | LRC404 | 120 | 2601.6 | 2500 | 124.9 | 104.1 | PASS |
| 59. | K010 | ARTEFAN | PA11971 | 120 | 2527.2 | 2500 | 121.3 | 101 | PASS |
| 60. | M020 | LUMAREN | 03121 | 120 | 2573.3 | 2500 | 123.5 | 102.9 | PASS |
| 61. | M019 | ARTEFAN | PA02931 | 120 | 2442.9 | 2500 | 117.3 | 97.7 | PASS |
| 62. | M018 | ARTEFAN | PA02811 | 120 | 2606.1 | 2500 | 125.1 | 104.2 | PASS |
| 63. | M013 | ARTEFAN | DJ0839G | 120 | 2511 | 2500 | 120.5 | 100.5 | PASS |
| 64. | M012 | ARTEFAN | PA034202 | 120 | 2451.8 | 2500 | 117.7 | 98.1 | PASS |
| 65. | M011 | LONART | LRC588 | 120 | 2572.9 | 2500 | 123.5 | 102.9 | PASS |
| 66. | M021 | LUMARTEM | QK91643 | 120 | 2438.9 | 2500 | 117.1 | 97.6 | PASS |
| 67. | M022 |  | HWE111217 | 120 | 2475.2 | 2500 | 118.8 | 99 | PASS |
| 68. | M023 | LONART | A1AFJ026 | 120 | 2507.3 | 2500 | 120.4 | 100.3 | PASS |
| 69. | M024 | LONART | K1AFJ018 | 120 | 2471.5 | 2500 | 118.6 | 98.9 | PASS |
| 70. | M025 | LONART | K1AFJ020 | 120 | 2511.8 | 2500 | 120.6 | 100.5 | PASS |
| 71. | M017 | CO-METHER | T13007 | 120 | 2495.1 | 2500 | 119.8 | 99.8 | PASS |
| 72. | M016 | LUMITER | NAA20167A | 120 | 2532.9 | 2500 | 121.6 | 101.3 | PASS |
| 73. | M015 | LUMITER | NAA20298A | 120 | 2450.9 | 2500 | 117.9 | 98 | PASS |
| 74. | M014 | COARTEM | KX118 | 120 | 2500.7 | 2500 | 120 | 100 | PASS |
